# Functional analysis of circulating tumour cells: the KEY to understand the biology of the metastatic cascade

**DOI:** 10.1038/s41416-022-01819-1

**Published:** 2022-04-28

**Authors:** Zahra Eslami-S, Luis Enrique Cortés-Hernández, Frédéric Thomas, Klaus Pantel, Catherine Alix-Panabières

**Affiliations:** 1 Laboratory of Rare Human Circulating Cells (LCCRH), University Medical Centre of Montpellier, Montpellier, France; 2 CREEC (CREES), Unité Mixte de Recherches, IRD 224–CNRS 5290–Université de Montpellier, Montpellier, France; 3 Institute of Tumor Biology, University Medical Centre Hamburg-Eppendorf, Hamburg, Germany

**Keywords:** Metastasis, Cancer stem cells

## Abstract

Metastasis formation is the main cause of cancer-related death in patients with solid tumours. At the beginning of this process, cancer cells escape from the primary tumour to the blood circulation where they become circulating tumour cells (CTCs). Only a small subgroup of CTCs will survive during the harsh journey in the blood and colonise distant sites. The in-depth analysis of these metastasis-competent CTCs is very challenging because of their extremely low concentration in peripheral blood. So far, only few groups managed to expand in vitro and in vivo CTCs to be used as models for large-scale descriptive and functional analyses of CTCs. These models have shown already the high variability and complexity of the metastatic cascade in patients with cancer, and open a new avenue for the development of new diagnostic and therapeutic approaches.

## Introduction

The multistep process by which a cancer cell migrates is called the metastatic cascade. Although the concept of metastasis is simple, the underlying mechanism is complex and varies significantly among individuals and/or cancer types [1, 2]. Similar to the evolution of the different species of living organisms, cancer is a process that adapts during the host lifespan. Somatic cell selection and evolution are the fundamental processes leading to malignancy and its numerous manifestations, including the propensity of cancer cells to migrate and establish metastases [3]. Therefore, there is not a single cellular mechanism that can explain how cancer cells migrate, as there is not a single strategy used by animals to survive and migrate.

Importantly, although circulating tumour cells (CTCs) are the main drivers of metastasis formation [4], most studies in cancer are done using primary or metastatic cancer tissues and tumour-derived cell lines. Consequently, most steps of the metastatic cascade have been neglected. This is not for lack of interest in these steps, but due to the absence of methods and models to study CTCs.

This has been changed by the development/improvement of technologies and methods to directly study CTCs, such as the CellSearch^®^ system (Menarini Silicon Biosystems, Inc), the PARSORTIX system (Angle), the ISET^®^ technology (Rarecells, Inc), and the ScreenCell technology, just to mention some. They have brought new opportunities to investigate CTCs and their association with clinical features [5–7]. For example, the enumeration of CTC-EpCAM^(+)^ in blood samples of patients with cancer has a prognostic significance, and in patients with metastatic breast cancer it can help to identify therapy failure earlier than the usual clinical monitoring [8, 9]. The clinical applications of CTC detection have already been reviewed elsewhere [7, 10].

However, CTCs are rare events in blood, and this hampers their identification and characterisation. In this context, the development of in vivo and in vitro models for CTCs are fundamental to identify the key mechanisms of metastasis formation. To this aim, specific methods to capture and to characterise viable CTCs (i.e. cells that can grow in vitro and be used in in vivo models) will allow the in-depth characterisation of metastasis-competent CTCs.

In this review, we summarise some of the most important advances in the functional study of viable CTCs in patients with cancer, as well as in vitro and in vivo models, and describe how some mechanisms of metastasis formation have been identified using these models. Moreover, we added an evolutionary ecology perspective to explain the complexity of the metastatic process.

## Detection of viable CTCs in patients with cancer

Many approaches for capturing and isolating CTCs have been and are currently developed based on the physical and biological characteristics of these cancer cells (summarised in Table 1). Most of these methods require the chemical or physical fixation of CTCs. Therefore, only few methods allow the isolation of CTCs that can then be expanded in vitro or in vivo (i.e. viable CTCs). These methods allow performing functional analyses of CTCs and thus, identifying the biological features of metastatic cells, and in some cases, even establishing CTC cultures in vitro.

**Table 1:** Different approaches for CTC enrichment and isolation.

Enrichment method	Cell viability	Culture possibility	Selection criteria	Advantages	Disadvantages	Ref
Acoustophoresis Chip	Yes	Yes	Cells are separated based on their acoustophoretic mobility, which is size-dependent, by exposing them to acoustic waves	Label-independent isolation, Acoustic pre-alignment and separation	No morphology confirmation, High contamination by WBCs	[73]
AdnaTest ®	No	No	Positive selection, immunomagnetic beads coated by antibodies against surface markers for CTC detection (antibodies selected in function of the cancer type)	High sensitivity, rapid processing, integrated mRNA isolation for CTC molecular characterisation; CTC analysis by multiplex RT-PCR with gene panels; commercial kit available for prostate, lung, breast and colon cancer	No morphology confirmation, High contamination by WBCs	[74]
ApoStream	Yes	Yes	Dielectrophoresis-based method to capture CTCs via multiple integrated electrodes that generate a non-uniform alternating electric field	Label-independent isolation, continuous flow, capture of viable cells, compatible with downstream biomarker assays	Low purity Long procedure Limited volume Cell electrical properties can be affected during the procedure Large number of parameters must be controlled simultaneously	[75]
CellSearch®	No	No	Positive selection, immunomagnetic enrichment (EpCAM^+^) and immunostaining for CTC detection	Adequate clinical evidence, automated enumeration, commercial availability automated system, reliable, reproducible, visual identification of cells	Limitation to detect EpCAM-negative CTCs, expensive, limited number of markers, subjective image evaluation, limited downstream analyses	[6]
Cluster-Chip	Yes	No	Microfluidic chip, based on size and asymmetry (triangular micropillars designed to immobilise CTC clusters). Relies on cell-to-cell junction strength	Label-independent isolation, the potential study of tumour-immune cell interactions, low shear stress that reduces on-chip residence time	Lack of biological characterisation and clinical significance; shear stress is needed to release most clusters from micropillars	[76]
CTC-iChip	Yes	Yes	Microfluid chip, negative enrichment by using hydrodynamic size-based sorting, plus magnetophoretic and immunostaining	Allows the sequential separation of different blood components through micropillar array, simplicity, enables the characterisation of CTCs with both epithelial and mesenchymal characteristics	Samples not suitable for sequencing, just high quality of RNA from untagged CTCs. Expensive, long set-up times, multiple steps	[77]
CTC-Chip	Yes	Yes	Microfluidic chip, CTC capture by anti-EpCAM antibody-coated posts	High detection rate, visual identification, usable for potential future functional analyses	EpCAM‐positivity dependent; EpCAM-negative CTCs not detected; requires clinical validation; time-consuming (1–2 mL/h)	[11]
DEPArray^TM^	Yes	No	Dielectrophoresis-based method	Designed for pure single CTC capture, recovery and manipulation of single cells; allows downstream gene analysis and sequencing	Long procedure Limited volume Cell electrical properties can be affected during the procedure Large number of parameters must be controlled simultaneously Needs pre-enrichment	[78]
EasySep	Yes	Yes	Negative selection immunomagnetic	Batch separation, does not bias the sample according to the selection markers	False-positive results due to CD45– endothelial cells, not high purity levels	[79]
EPISPOT	Yes	Yes	Combination of CD45 depletion (RosetteSep) and density gradient for CTC enrichment, coated well plate for detection of viable CTCs	Captures and detects viable CTCs, discriminates between viable and apoptotic CTCs using protein secretion. High sensitivity and specificity, allows CTC quantification	Proteins must be actively secreted. Limited number of markers. Unbiased enrichment independent of the CTC/DTC phenotype	[14]
EPIDROP	Yes	No	Combination of negative enrichment using CD45 depletion (RosetteSep) and density gradient for CTC enrichment. Immunostaining for CTC detection through microfluid chip	Simultaneous proteomic and secretome analyses of single viable CTCs: oncogram. High sensitivity and specificity. Automatic detection of positive events using an appropriate software	Prototype development still in progress, limited number of markers, not possible to recover CTCs from chip	[51]
GILUPI CellCollector	Yes	No	EpCAM-coated wire placed intravenously in patients for CTC collection	Can process large volumes of blood. No need of blood sampling. Can isolate rare CTCs at early cancer stages. Increases probability to detect CTCs in vivo	Captures only EpCAM-CTCs. May be not accepted by patients. Time-consuming, CTCs cannot be released from the wire	[80]
Herringbone CTC-Chip	Yes	No	Microfluid chip (surface-capture device)	CTC cluster detection, short procedure, the possibility of downstream analyses, low shear	Trypsin needed to detach CTCs from the Chip	[12, 81]
ISET^TM^	No	No	Filter-based (size/ deformability) isolation and enrichment	Easy and rapid processing, label-independent isolation, sensitivity threshold of 1 CTC/mL of blood; cluster detection, isolation of EpCAM-negative CTCs, FISH, DNA/RNA analysis on CTCs	Size‐dependent (may miss cells <8 μm in size), blood cells need to be fixed, false-positive results, low recovery and purity	[82]
IsoFlux CTC	Yes	-	Microfluidic system with controlled flow and immunomagnetic capture bead system (positive enrichment)	Capacity to detect genetic alterations, identify both epithelial and mesenchymal CTCs	Semi-automated, 50% of capture rate, time-consuming	[83]
MagWIRE	Yes	No	Magnetic wire for intravascular retrieval and enrichment using antibody-coated magnetic particles	Large-scale CTC capture in vivo, small diameter, very fast processing, completely self-contained, flexible. Analysis by qPCR	Low efficiency, captures only EpCAM^+^ CTCs. Possible systemic exposure to excess iron. May not be accepted by patients. Long procedure	[84]
Metacell^TM^	Yes	Yes	Gentle flow within the size-based separation	Potential study of cytomorphology and molecular diagnosis, fast processing	Over-collection of other blood cells Low specificity	[85]
OncoQuick^TM^	Yes	Yes	Combined density-based gradient centrifugation and filtration by integrating a porous barrier	Porous membrane prevent mixing, simple, inexpensive, increased depletion of mononuclear cells	Relative low yield and enrichment	[86]
Parsortix™	Yes	Yes	Size/ deformability isolation and enrichment	Label-independent isolation, viable cells released by reversing flow Possibility of downstream analysis High specificity	Difficulty to differentiate CTCs and WBCs Low specificity	[87]
RosetteSep^TM^ CTC	Yes	Yes	Immunodensity- negative selection using an antibody cocktail	Easy and quick procedure, chip, isolation of viable CTCs,	Antibody-labelled might alter cell density, Low purity	[88]
ScreenCell^®^ Cyto	Yes	Yes	Filter-based size-exclusion separation and enrichment of CTCs	Potential study of cytomorphology and molecular biology, fast processing, Post-capture analysis	Over-collection of other blood cells Low specificity	[89]

In this table are summarised the different methods and strategies for CTC enrichment and isolation.

Viable CTC enrichment can be achieved by exploiting their physical properties, for example by density gradient centrifugation using Ficoll-Paque^TM^ (GE Healthcare, Chicago, IL, USA), Lymphoprep^TM^ (STEMCELL Technologies, Vancouver, Canada), or the OncoQuick^®^ gradient system (Greiner Bio-One, Kremsmunster, Austria). These methods allow the label-free enrichment of CTCs and maintaining their viability for subsequent in vitro culture. However, centrifugation might lead to significant CTC loss, and the total yield of highly pure CTCs is low (mixed populations containing both leucocytes and CTCs). Therefore, other detection techniques (e.g. immunofluorescence, immunohistochemistry, FISH) must be subsequently used to identify/purify and to specifically characterise CTCs. To increase CTC recovery rate without any bias, some methods (e.g. the RosetteSep^TM^ CTC method) combine immunoaffinity negative selection with density gradient centrifugation. Unwanted cells are targeted by tetrameric antibody complexes and depleted. CTCs can then be collected near the plasma-density medium interface.

Alternatively, microfluidic systems can be used in which cells are trapped inside the system for characterisation or/and expansion. Several microfluidic systems have been developed in the last years for CTC purification and characterisation. For instance, in the microfluidic-based CTC-chip technology, micro-sized posts are chemically functionalized with anti-EpCAM antibodies [11]. However, due to the micro-post structure complexity, the scaling up of this system for high-throughput production and large-scale clinical applications is challenging. To improve CTC capture, high-throughput surface-capture based devices have been developed. For instance, in the herringbone-chip (HB-Chip) technology, the chip wall surfaces are patterned with herringbones to create micro-vortices that maximise collisions between the target cells and the antibody-coated walls. Cell viability is ~95%, but a trypsinization step is needed for CTC release, and this might affect the expression of receptors on CTCs [12]. CTC-iChip is a microfluidic immunomagnetic-based CTC isolation device that can sort epithelial and non-epithelial cancer cells using a label-dependent or a label-free process (micropillar array, hydrodynamic size-based sorting and magnetophoretic). This allows the removal of tagged hematopoietic cells, and the efficient capture and isolation of viable CTCs that may be cultured. However, it has long set-up times. All these methods allow the in vitro expansion of CTCs, to different degrees; however, additional steps are needed to demonstrate the viability of the captured cells.

**Fig. 1:**
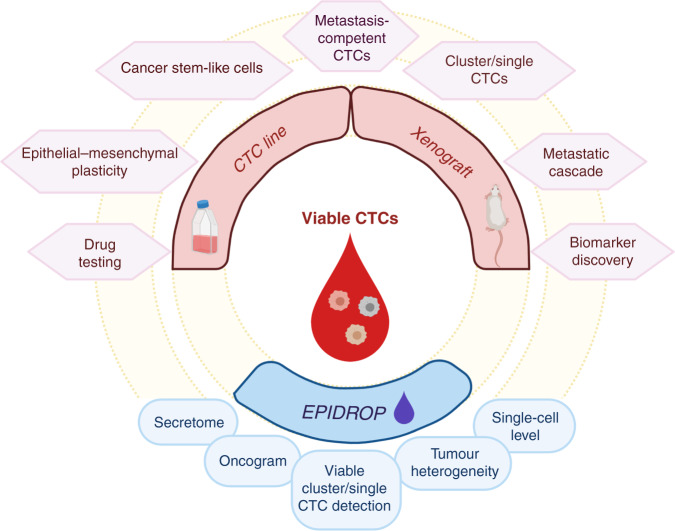
Functional study of CTCs. The enrichment and subsequent expansion of CTCs have many translational applications. The analysis of these cells as CTC lines or xenografts can expand our comprehension of the metastatic process and lead to the identification of new biomarkers and/or pathways to propose potential new therapies against cancer. Moreover, an innovative functional approach, called EPIDROP, can help to study single viable CTCs directly from a blood sample. The analysis of viable CTCs might open the opportunity to test drugs in real time in vitro to evaluate therapy sensitivity/resistance: the oncogram.

Therefore, other methods have been developed to directly assess CTC viability in order to easily identify metastasis-competent CTCs in fewer steps. The Epithelial Immuno-SPOT (EPISPOT) assay was introduced for the functional study of viable CTCs. In this method, CTCs are cultured on a membrane coated with antibodies for a short time, and then the proteins secreted, shed, or released by viable CTCs are detected. This method has been validated in the clinic for breast [13] prostate [14], melanoma [15], head and neck [16] and colorectal cancer [17]. To increase the EPISPOT sensitivity and specificity, a new optimised assay, called EPISPOT in a DROP (EPIDROP), is currently under development (analytical and clinical validations). This innovative microfluidic system will provide a unique diagnostic medical device for functional studies of viable CTCs. In the EPIDROP assay, a panel of surface biomarkers is used to increase CTC capture yields after a negative enrichment step (leucocyte depletion). This assay can identify also CTCs without epithelial features (i.e. EpCAM-negative) and very small CTCs. The EPIDROP assays may allow monitoring cancer progression and drug testing at the single-cell level for each individual patient, to define a personalised “oncogram”. Currently, three clinical trials are recruiting patients with cancer (blood sample collection) to evaluate the EPIDROP potential for different clinical applications (PROLIPSY, NCT04556916, for the early detection of prostate cancer; EPIDROP, NCT04581109, in patients with metastatic prostate cancer; and ALCINA, NCT02866149, breast and lung cancer and immunotherapy) (Fig. 1).

Another microfluidic-based immunomagnetic isolation method has been used for the functional study of viable CTCs in lung cancer. This method exploits the unique metabolomic activity of CTCs to identify live CTCs. Specifically, cancer cells or CTCs are distributed in the chip microwells and incubated with a fluorescent glucose analogue. Due to the Warburg effect, live cancer cells/CTCs show high uptake of this analogue and can be observed using fluorescence microscopy [18].

## Strategies to catch metastasis-initiator CTCs (in vivo and in vivo systems)

The development of (short or permanent) CTC lines and xenografts derived from CTCs can provide novel functional insights into these cells. These models are useful for identifying signalling pathways unique to metastasis-competent CTCs, monitoring different patterns of drug resistance in individual patients, and discovering new CTC markers, and also offering insights into the metastatic process.

### In vitro expansion of CTCs

Due to CTC's low frequency, expanding CTCs is a requirement for their functional study. Importantly, CTC expansion will allow focusing only on the more aggressive metastasis-initiator CTCs because only this specific CTC subgroup has self-renew potential. In addition, the in vitro expansion of CTCs offers the attractive possibility to assess the molecular profile and drug sensitivity/response to therapy of these cancer cells. To date, some research groups managed to culture CTCs from different cancer types for few days (from 3 to 14 days up to 40 days), and only few groups managed to establish long-term CTC cultures. Table 2 summarises information on how to isolate and culture CTCs.

**Table 2:** In vitro culture of CTCs isolated from blood samples of patients with cancer.

Cancer type	Isolation methods	Culturing conditions	Time in culture	Ref
Breast cancer	Multiparametric FACS analysis	First 7 days: DMEM/F12 with 5 mg/ml insulin, 0.5 mg/ml hydrocortisone, 2% B27, 20 ng/ml EGF, and 20 ng/ml FGF-2. From day 8: EpiCult-C medium with 10% FBS and 1% penicillin/streptomycin at 37 °C, 5% CO_2_.	Short term	[19]
Breast cancer	CTC-iChip	Serum-free medium supplemented with EGF and bFGF (8) in hypoxic conditions (4% O_2_).	Long term	[21]
Breast cancer	Laser-ablated microwell-based method	High-glucose DMEM with 10% FBS and 1% penicillin–streptomycin, at 37 °C in 5% (v/v) CO_2_ and 1% O_2_ in humidified conditions.	Short term	[22, 90]
Breast cancer	Ficoll-Paque density gradient and CD45 RosetteSep negative selection	RPMI-1640, 10% FCS) 1% penicillin–streptomycin, 1% l-glutamine, 1% insulin-transferrin-selenium-A supplement (100×) liquid, 10 ng/ml FGF-2, 50 ng/ ml EGF, 0.1 lg/ml hydrocortisone, and 0.2 lg/ml cholera toxin, at 37 °C, 5% CO_2_.	Long term	[91]
Colon cancer	Ficoll-Paque density gradient and CD45 RosetteSep negative selection	RPMI-1640 with 2% FBS, EGF and FGF-2, insulin-transferrin-selenium supplement, l-glutamine.	Long term	[25, 27]
Non-small cell lung cancer	Herringbone-chip	7 days on a chip in a mixture of cancer-associated fibroblasts-GFP, collagen I and Matrigel, at 37 °C, 7.5% CO_2_, Then in culture plate with RPMI complete medium (10% FBS and 1% penicillin/streptomycin).	Short term	[92]
Small cell lung cancer	Ficoll-Hypaque density gradient	﻿Serum-free RPMI-1640 medium supplemented with insulin, IGF-1, transferrin.	Short term	[32]
Prostate cancer	Ficoll-Paque density gradient and CD45 RosetteSep negative selection	DMEM/F12 medium, with EGF, bFGF, FGF10, R-spondin 1, DHT, B27, nicotinamide, A83-01, SB202190 and Y27632, in Matrigel.	Long term	[33]

*FBS* foetal bovine serum, *bFGF* basic fibroblast growth factor, *FGF* fibroblast growth factor, *DHT* dihydrotestosterone, *EGF* epidermal growth factor, *IGF-1* insulin-like growth factor 1.

In this table are summarised some of the studies where CTC in vitro expansion have been reported.

#### Breast cancer

The establishment of CTC cell lines from blood samples of patients with metastatic breast cancer was reported for the first time in 2013. Zhang et al. described the existence of a CTC subpopulation that does not express the EpCAM marker, and proposed that metastasis formation might not always require CTC-EpCAM^(+)^ [19]. In this study, both in vitro and in vivo models were essential to identify the CTC subpopulation with metastatic capacity. Cancer dormancy also might reduce the expression level of epithelial markers in CTCs [20]. Yu et al. could expand CTCs from blood samples of patients with oestrogen receptor (ER)^(+)^ breast cancer for more than 6 months by using hypoxic and non-adherent culture conditions [21]. Interestingly, the individual mutational profile of the obtained CTC lines showed different drug sensitivity patterns [21]. In another study on CTC clustering, Khoo et al. used laser-ablated microwells and leucocytes as co-culture components to mimic the tumour microenvironment [22]. During chemotherapy, cluster formation diminished, reflecting the patient’s response to treatment. Jakabova et al. cultured CTCs from patients with breast cancer at different stages. Although they did not observe any significant difference among subgroups, CTC occurrence was highest in the group undergoing surgery and in the groups before the start of neoadjuvant and adjuvant treatment [23]. Recently, Koch et al. established a CTC-EpCAM^(+)^ line derived from patients with metastatic ER^(+)^ breast cancer and endocrine therapy resistance. Copy number alteration (CNA) profiling indicated high concordance between the original CTCs directly captured from the blood sample and the established CTC line. In addition, functionally relevant mutations in the *MAP3K1*, *MAP3K6*, *NF1* and *PIK3CA* genes were detected in the CTC line, primary tumour, and metastasis site. This indicated that the CTC line mirrors the situation in the ER^(+)^ breast cancer and thus can provide novel insights into the biology and therapeutic response in this cancer subtype [24]. Then, the metastatic potential of this CTC line was verified by injection into the mammary glands of female immunodeficient mice. Tumour burden increased steadily over time until sacrifice and spread to the same metastatic sites as in the patient.

#### Colorectal cancer

The first stable CTC line from a patient with metastatic colon cancer was obtained by Cayrefourcq et al. in 2015 [25]. This cell line (CTC-MCC-41) presents epithelial features with stem cell-like characteristics and the main genomic characteristics of the primary tumour and lymph node metastases [25]. In addition, the differential expression of genes involved in energy metabolism and DNA repair could be detected [26]. Moreover, eight additional cell lines could be established and characterised from the same patient at different stages of clinical management: during treatment and during cancer progression. This unique series of nine CTC lines highlight the selection mechanism of treatment-resistant clones with specific phenotypes that drive disease progression [27, 28].

#### Lung cancer

Zhang et al. developed a novel in situ capture and 3D co-culture model for CTC expansion that simulates the tumour microenvironment. They successfully expanded CTCs for a short period of time and compared their genome profiles to those of the primary tumours [29]. The same procedure was used to expand CTCs from a single patient with ALK-positive lung cancer to estimate in real time the drug resistance profile caused by the ALK rearrangement by testing different drugs in vitro. Que et al. generated a CTC line from CTCs of a patient with stage IIa non-small cell lung cancer (NSCLC) using a one-step microfluidic-based immunomagnetic separation approach [30]. The high and low expression levels of CXCL5 and CX3CL1 in CTCs, respectively, suggest a role of these proteins in metastasis formation. The metastatic potential of this CTC line was confirmed in a mouse model [30].

In small cell lung cancer (SCLC), Hamilton et al., obtained a CTC line by density gradient centrifugation with a leucocyte depletion step for CTC enrichment. They showed that the CTC line expresses EMT markers in different proportions that might reflect its aggressiveness. Conversely, this line does not express stem cell markers related to the suppression of epithelial markers [31, 32]. CTC spheroids could be formed, but cells remained viable only for a short time. This CTC model was used for drug sensitivity testing. Indeed, CTC culture may be useful for biomarker identification and drug sensitivity testing to provide a “real-type” prediction of the response to treatments.

#### Prostate cancer

The only example of successful long-term culture of CTCs obtained from patients with prostate cancer was reported by Gao et al. in 2014 [33]. They used a 3D organoid system that allows better capturing the tumour-specific characteristics [33]. CTCs were enriched by using negative selection, followed by red and white blood cell depletion by density gradient. This CTC line represented the prostate cancer molecular diversity. No change in gene expression was observed between the CTC line and the primary tumour. CTC capacity to generate tumours was confirmed in mice [33].

More recently, diagnostic leukapheresis (DLA) was used to obtain higher numbers of CTCs from patients with metastatic prostate cancer to explore their capacity to form organoids. This technique has been proposed as a platform for ex vivo treatment modelling. Mout et al. hypothesised that DLA can increase significantly the number of collected CTCs and consequently, increase the chance to generate CTC-derived organoids. However, major challenges are the high blood volume needed and the excess of leucocytes present in the sample. In this study, organoids could be produced from 35% of the collected samples, including one long-term CTC culture (EMC-PCa-41) that harbours similar drug resistance as the patient’s tumour. Most organoid cultures could be maintained only for 6–8 weeks [34].

### CTC-derived xenografts (CDX)

Due to the difficulties in establishing in vitro CTC lines, other methods, such as xenograft models, have attracted great attention. Compared to CTC lines, this model mimics the cancer molecular complexity and heterogeneity and offers the possibility to evaluate personalised therapies in vivo [35] (Table 3). Tumours generated from CTC injection (CTC lines or CTCs directly isolated from patients) should show a similar phenotype and genotype as metastatic cancer. CTC-derived xenograft (CDX) models allow the identification of CTC subpopulations by sorting and characterising them from the different sites of tumour formation after CTC injection.

**Table 3:** In vivo CTC-derived xenograft models.

Type of cancer	Isolation method	Injection procedure	Main results	Ref
Breast cancer	RosetteSep and FACS (PI- CD45-EpCAM + )	-Dilution in Matrigel - Injection in femoral medullar cavity	- Drug sensitivity not evaluated - Specific CTC MIC signature: EpCAM+CD44 + MET + CD47 + - decreased progression-free survival of patients with CD44 + , CD47 + and MET + CTCs	[36]
Breast cancer	Density gradient centrifugation: Histopaque®	- Dilution in Matrigel - Subcutaneous injection	- Drug sensitivity not evaluated - CDX model was representative of the primary tumour features: ER- PR- pan-CK + aECAD+ - identification of MELK as a prognostic marker of TNBC - WNT pathway upregulation as a potential therapeutic target in TNBC	[37]
Breast cancer	Multiparametric FACS (CD45-/CD34-/CD105-/CD90- CD73-)	- Intracardiac injection in aseptic conditions	- Distinct transcriptomic signatures between CTCs from primary tumour site in patients and the corresponding model - Survival analyses of transcriptome signature - Identification of 597 genes related to liver metastasis in TNBC	[20]
Small cell lung cancer	Ficoll-Paque density gradient and CD45 RosetteSep negative selection	- Dilution in Matrigel - Subcutaneous injection	- CDXs represent clinical SCLC - Drug sensitivity was evaluated and CDX mimicked the donor’s response to chemotherapy - CDX tumours reflect CTC genomic profile	[39]
Small cell lung cancer	Ficoll-Paque density gradient and CD45 RosetteSep negative Selection CTC-iChip	- Dilution in Matrigel - Subcutaneous injection	- Drug sensitivity evaluated - Correlation of MYC signatures with drug resistance by transcriptomic analysis - CDX mirrors the patient’s cancer progression	[40, 93]
Non-small cell lung cancer	Ficoll-Paque density gradient and CD45 RosetteSep negative selection	- Dilution in Matrigel - Subcutaneous injection	- Importance of mesenchymal CTCs with tumorigenic capacity - Absence of CTC-EpCAM^(+)^ is not a limitation for metastasis formation	[41]
Prostate cancer	DLA/R Ficoll-Paque density gradient and CD45 RosetteSep negative selection	- Dilution in Matrigel - Subcutaneous injection	- Same genome characteristics in CTC, patient tumour, and CDX - Tumorigenic CTCs with acquired CRPC-NE features - Genomic alternations in CDX models	[43]
Melanoma	Ficoll-Paque density gradient and CD45 RosetteSep negative selection	- Dilution in Matrigel - Subcutaneous injection	- Drug sensitivity and patients’ response to treatment were evaluated - Concordance in SNV profiles - CTCs have similar tropism as the patient’s tumours	[44]

*FACS* fluorescent-activated cell sorting, *CDX* CTC-derived xenograft, *TNBC* triple-negative breast cancer, *SNV* single-nucleotide variant, *NE* neuroendocrine, *MIC* metastasis-initiator cells.

This table summarises the different attempts to establish CTC-derived xenografts.

The first CDX model was described by Baccelli et al. in 2013 [36]. They injected CTCs from patients with metastatic breast cancer into the femoral medullar cavity of immunodeficient mice that developed bone, lung and liver metastases. CDX developed in mice only when high numbers of CTCs could be isolated from the patient’s blood sample. Moreover, the identification of CTCs that express CD44, CD47 and MET was strongly correlated with decreased progression-free survival in these patients [36]. Another study reported the establishment of a CDX model from a patient with advanced metastatic triple-negative breast cancer (TNBC) and high CTC number. CTCs were injected subcutaneously into mice and tumours could be detected after 5 months. Moreover, samples were collected at metastatic sites, thus allowing the real-time characterisation of tumour samples, CTCs, and CDXs [37]. To decipher the genomic/transcriptomic properties of TNBC liver metastases for potential therapeutic targeting, Vishnoi et al. established CDX by injection of CTCs from patients with TNBC in immunodeficient mice. The distinct transcriptomic signature of the CTCs isolated from the patients was maintained in the corresponding CDX model. Moreover, the authors discovered a CTC signature of 597 genes related to CTC-driven liver metastasis formation that provides information on the mechanisms of TNBC recurrence in liver [38].

Hodgkinson et al. evaluated the tumorigenicity of CTCs from patients with advanced metastatic SCLC. They could derive CDX only from patients’ blood samples with high CTC number (>400) [39]. Drapkin et al. used an automated microfluidic chip for viable CTC isolation from blood samples of patients with SCLC to generate CDX models in nude mice (38% of efficiency versus 89% for patient-derived xenograft models using tumour biopsies). The transcriptomic analysis showed that the MYC signature was strongly associated with drug resistance. These models also mimicked the patients’ changes in drug sensitivity, highlighting the potential usefulness of CDXs for SCLC management [40].

Morrow et al. presented a clinical case study concerning a patient with metastatic NSCLC. They could not obtain any CDX at baseline (before treatment), but only after radiotherapy using CTCs that showed a mesenchymal phenotype. This study suggests that the absence of CTC-EpCAM^(+)^ is not a limitation for metastasis formation. It also highlights the importance of investigating CTCs undergoing EMT for establishing CDX models from metastatic cancers [41].

Rossi et al. evaluated the capacity of CTC-EpCAM^(+)^ from patients with metastatic prostate cancer to generate tumours in mice. At ~10 months after injection, they detected human CTCs in blood samples from all injected mice, but not tumours. Even in mice injected with a very small number of CTCs, single CTCs could be detected in peripheral blood, spleen and bone marrow [42]. Another study reported the generation of a CDX model and a CDX-derived cell line from a patient with castration-resistant prostate cancer (CTCs collected by DLA) [43]. The CDX and the CDX-derived cell lines conserved 16% and 56% of the mutations detected in the primary tumour and collected CTCs, respectively, and 83% of the primary tumour CNA. Overall, CDX genomic characterisation revealed some changes, such as TP53 mutations, RB1 loss, and PTEN deficiency [43]. Girotti et al. showed that melanoma CTCs are tumorigenic with a similar tropism as the patient’s tumour. They reported a success rate of 13% for CDX establishment. The CDX tumour was detected 1 month after CTC implantation and was sustainable in secondary hosts [44].

CDX-derived cells, in which in vitro and in vivo approaches are combined, can be used for functional studies to test new therapeutic approaches, identify novel biomarkers and understand the drug response mechanisms. So far, this model has been only used for CTCs isolated from patients with SCLC, mostly to evaluate drug response. Lallo et al. showed that CDX models accurately reproduce the genetic characteristics, pathology, and treatment response of the patient [45]. These CDX models have been used to test the efficacy of olaparib and/or AZD1775 (PARP and WEE1 inhibitor, respectively) in CDX-carrying mice. In cell lines derived from these CDX, mutations in the *PALB2* gene are linked to resistance to these inhibitors [45].

## Biology of metastasis-competent CTCs

CTCs originate when cancer cells are released from the main (primary or metastatic) tumour mass; however, there are not all equal in their capacity to colonise distant tissues and form metastatic tumours. Thus, CTCs can be divided into two groups in the function of their metastatic capacity: CTCs that gain migratory features and can reach actively the general circulation, but (1) are not metastasis-competent and (2) CTC that are metastasis-competent. In the second category, some variability also exists, yielding a range of more or less metastasis-competent CTCs.

In the clinic, metastasis-competent CTCs have the highest impact in disease progression because they can successfully complete all the metastatic cascade steps. It is well established that this CTC subgroup represents a minimal part of the total number of CTCs in blood. Indeed, Fidler et al. demonstrated in a seminal work that less of 0.01% of cancer cells injected in in vivo models could generate metastatic tumours [46, 47]. Even this percentage might not reflect the true potential of CTCs to form metastases in patients. In vitro expanded CTCs might better reflect this specific CTC subpopulation that is already an extremely rare event in blood [4]. Indeed, to grow in vitro, a CTC must be able to proliferate in a completely different environment.

Metastasis-competent CTCs do not present universal characteristic, probably because cancer is a highly heterogenous disease. Likewise, the metastatic mechanisms are very different in function of the cancer type, patient and organs. Moreover, these mechanisms evolve during treatment and/or disease progression. One example of this is the high variability in CTC-EpCAM^(+)^ detection between SCLC and NSCLC. Although both cancer types originate from the same organ, the mean CTC-EpCAM^(+)^ number differs drastically between SCLC and NSCLC [48, 49], suggesting different metastatic mechanisms.

Despite this heterogeneity, all metastasis-competent CTCs can self-replicate in a similar way as stem cells [50]. Therefore, CTCs that display these features are considered as cancer stem-like cells (CSCs) or circulating cancer stem-like cells (CCSCs). However, different cancer types present distinct stem cell marker expression profiles. EpCAM is overexpressed in carcinomas and has been proposed to be a stem cell marker [51]. Indeed, EpCAM expression is reported in most studies on in vitro expanded CTCs. Zhang et al. were the only to detect a CTC-EpCAM^(−)^ subpopulation in their short-term in vitro expanded CTCs. This subset also expressed ALDH1, another stem cell marker, and could form distant tumours after injection in immunodeficient mice [19]. Moreover, other cancer types of non-epithelial origin express different stem cell markers, for instance CD34 in leukaemia [52]. These variabilities might mirror the high diversity of metastatic mechanisms.

Despite this variability, EpCAM expression in CTCs is important during the metastatic process. For instance, CTC-EpCAM^(+)^ enumeration in blood predicts the outcomes of therapy in breast cancer [8], and long-term in vitro expansion of CTCs and CTC lines have been obtained only with CTC-EpCAM^(+)^ CTCs [51].

EpCAM expression is associated with an epithelial phenotype, but these cells can migrate to distant sites. To achieve this, cancer cells must go through the epithelial–mesenchymal transition (EMT), which is a mechanism observed during embryogenesis and wound healing [53]. EMT occurs when adherent junctions between epithelial cells are dissolved. Then, epithelial cells gain mesenchymal features and can migrate, but their proliferation rate is reduced. To maintain a balance between migration and proliferation, metastasis-competent CTCs do not become completely mesenchymal, but acquire just enough mesenchymal features to detach from the original tumour. The term “partial EMT” has also been suggested to better express this balance [54]. Moreover, EMT molecular mechanisms are not the same in all cancer or cell types. For example SLUG, an essential transcription factor in the EMT of breast CSCs, is not detected in CSCs derived from single-layer epithelium [55]. These discrepancies between CSCs might also be related to their molecular profile and specific mutations; for example, the presence of the KRAS mutation G12D facilitates EMT in pancreatic cancer cell lines [56].

EMT is also associated with therapy resistance. It has been suggested that EMT may be a mechanism of survival in unfavourable conditions. As chemotherapy agents affect mainly highly proliferative cells, CSCs acquire mesenchymal features, proliferate less and move out in search of more favourable conditions (or time) where they can regain their epithelial phenotype and behaviour [57]. For example, it has been shown that in patients undergoing treatment for breast and lung cancer, the expression of EMT-associated genes correlates with resistance to therapy and disease progression [58–60]. In agreement, in different cancer types, the number of CTC-EpCAM^(+)^ is reduced after the first chemotherapy cycles [4, 28, 61]. This could be due to a reduction of the total number of cancer cells or due to EMT increase. Once the tumour clone develops chemoresistance mechanisms for survival, the number of CTC-EpCAM^(+)^ increases again, reflecting therapeutic failure. Cayrefourcq et al. described the molecular and clonal evolution in response to chemotherapy in their nine colon CTC lines obtained from a patient with metastatic CRC during progression and therapeutic failure [27]. Although all these CTC lines show clear stem cell features, only those obtained after progression express chemoresistance-associated markers. Moreover, Mani et al. reported a correlation between EMT and stemness by showing that in vitro immortalised non-tumoral mammary cell lines express stem cell markers after EMT induction [62]. Altogether, these observations support the intrinsic association of stemness and EMT and explain how this may limit treatment efficacy.

Moreover, stem cells and also cultured CTCs can form spheroids in vitro, particularly in hypoxic conditions [21]. This might be related to CTC capacity to form clusters and microemboli in blood [22, 25]. CTC clusters present a specific hypomethylation pattern related to stem cell features. For instance, Gkoutela et al. found that in breast cancer, transcription factor binding sites of stemness genes, such as *OCT4*, *NANOG*, *SOX2* and *SIN3A*, are hypomethylated in CTC clusters from blood samples [63].

Recently, Donato et al. showed that in mouse models xenografted with fluorescently labelled breast cancer cells, CTC clusters are released from hypoxic tumour regions. Conversely, when pro-angiogenic conditions are induced, single CTCs are released, and metastasis formation is reduced [64]. In agreement, hypoxia in breast cancer is associated with higher metastatic formation and chemoresistance. Similarly, in patients with cancer, CTC cluster number predicts a poorer prognosis compared with single CTC number. Moreover, CTC clusters can include also other cells from the tumour microenvironment [65]. Altogether, this might influence the response to anti-VEGF therapy [64]. This is a very clear example of the high adaptability of tumours and of the metastatic process.

However, the metastatic potential of CTC clusters might not be the same in all cancer types and might evolve to adapt to therapies. Metastatic competent CTCs might be just the result of clonal evolutive adaptations of the malignant cells or groups of cells (see for instance [66]) to survive, and as such these mechanisms are probably very variable and will be clearly related to environment pressures, genetic and epigenetics clues. For instance, the release of CTCs to the blood is suggested to have a circadian rhythm [67, 68].

## Conclusions

**Fig. 2:**
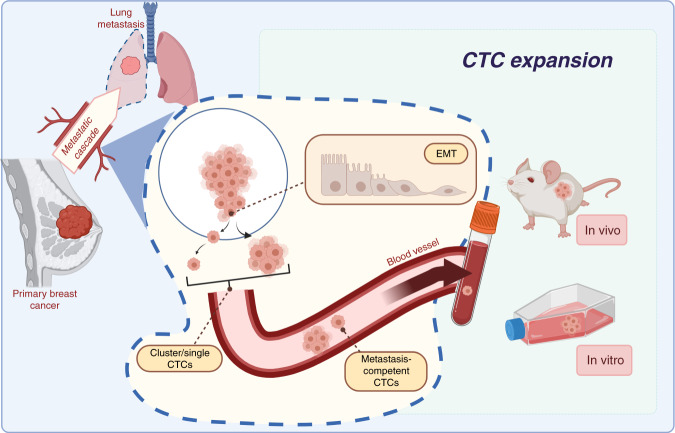
CTC models. The in vitro and in vivo expansion of CTCs allow the thorough characterisation of the liquid phase of the metastatic cascade. Although not all CTCs are metastasis-competent, CTCs with specific traits (e.g. stemness) that facilitate metastasis formation can grow in vitro or in immunodeficient mice. Thus, CTC models allow selecting metastasis-initiator CTCs and better understanding of the biology of these CTCs that are the basis of cancer progression.

CTCs are one of the circulating biomarkers of liquid biopsies that reveal information about the biology of the metastatic cascade in patients with cancer. Their potential is not limited just to be a diagnostic or therapeutic biomarker and it is important to thoroughly study metastasis-competent CTCs because this knowledge might help to develop new therapeutic strategies to eradicate specifically these bad CTCs. The in vitro and in vivo studies using CTCs reviewed in this article could be the “holy grail” to characterise the metastatic process in flagrante (Fig. 2). Although only few groups managed to generate CTC lines or developed robust methods to expand CTCs, their discoveries have added significant knowledge on the metastatic cascade the underlying mechanisms of which may be more complex than the situation in experimental models. In addition, CDX, particularly from patients with very advanced disease who have exhausted all conventional treatment options, might be an interesting alternative non-invasive strategy because obtaining tissue biopsies for testing/developing new treatment strategies may not be feasible in these patients. Indeed, CDX can provide novel insights into the biology of malignant cells in that specific patient (i.e. patient-specific model) to provide practical information on the therapeutic response.

Nevertheless, CTC analysis alone is not sufficient to understand metastatic progression, particularly due to the crucial contribution of the microenvironment of metastatic organs. For example, the interaction with other blood cells (e.g. neutrophils) can help CTCs to survive their dangerous travel in the blood [65]. Moreover, after extravasation at a distant site, their survival will depend on the existing environment conditions (e.g. low oxygen levels in bone marrow) [69] and on the presence of immune cells that attack the invaders [70]. Including in future studies both CTCs and circulating host cells will open a whole new dimension for the study of metastasis. Interestingly, when we tried to determine whether there is *one* key step in the metastatic cascade by using a modified Drake equation, our simulation predicted that the most critical parameter is the survival duration of CTCs [71]. This suggests that therapies targeting CTC survival in the vascular system may significantly reduce the risk of metastasis.

Finally, efforts should now focus on developing a standardised and robust method to expand CTCs from different cancer types. Novel approaches to increase CTC capture rate play an important role [72], and projects have been started by different consortia, such as the European Liquid Biopsy Academy (ELBA), and the European Liquid Biopsy Society (ELBS, www.elbs.eu). The establishment of CTC lines and the development of CDX represent a great opportunity to decode the metastatic cascade and test cancer drugs that specifically target CTCs [24].

## Data Availability

Not applicable.
